# *In vitro* quantitative comparison of erosive potential of infant mouthwashes on glass ionomer cement

**DOI:** 10.4317/jced.54314

**Published:** 2018-03-01

**Authors:** Aline-Bastos da Silva, Nayre-Maria-Lauande Rapôso, Isabella-Azevedo Gomes, Letícia-Machado Gonçalves, Marco-Aurélio-Benini Paschoal

**Affiliations:** 1 Undergraduate Student of the Dentistry, Universidade CEUMA, São Luís, MA, Brazil; 2 Graduate Student of the Master Program in Integrated Dentistry, Universidade CEUMA, São Luís, MA, Brazil; 3 Professor of Dentistry, Universidade CEUMA, São Luís, MA, Brazil; 4 Professor of Program of Integrated Dentistry, Universidade CEUMA, São Luís, MA, Brazil

## Abstract

**Background:**

The widespread use of mouthwashes, specially in children, is a concern, since the long-term use may modify the topography of dental materials. However, this process still unclear regarding the wear related to infant mouthwashes on glass ionomer cement. Thus, the purpose of this investigation was evaluate the erosive potential of infant mouthwashes on glass ionomer cement specimens.

**Material and Methods:**

Forty round-shaped specimens were divided into 4 groups (N=10) and submitted to erosive cycling for 15 days, being exposed 2X/day in the following children’s active agents mouthwash solutions: G1- cetylpyridinium chloride, G2- xylitol and triclosan and G3 - Malva sylvestris and xylitol. Prior to cycling, the specimens were submitted to the surface roughness measurement. After erosive cycling, the specimens were reanalyzed, and calculated the increase of roughness (∆Ra). Additionally, it was adopted distilled water as a negative control (G4). As an extra analysis, the mouthwashes had their pH values measured. The results were submitted to T-test and ANOVA followed by Tukey test at 5%.

**Results:**

In relation to pH values, G2 presented the most acidic pH value (pH = 6.83) in comparison to other substances. Regarding the comparison of the final roughness values (R) among the groups, it was verified that the mouthwashes showed significant roughness increase in comparison to control group, especially to G3 group (Rf = 1.67 ± 0.14) as well the ΔRa values with statistical difference in comparison to distilled water. Still, with exception of control group outcome, an increase of roughness of each mouthwash was verified after the studied period.

**Conclusions:**

Active agents present in infant mouthwashes were capable of roughness increased of glass ionomer cement surface, demonstrating an erosive potential of this material largely used in pediatric dentistry.

** Key words:**Dental erosion, dental cements, mouthwash.

## Introduction

With the increasing concern about more effective oral hygiene habits, particularly as regards to the child population, the use of chemical control agents (e.g., mouthwashes) has been adopted to complement toothbrushing and dental flossing (1).

An investigation have shown that when enamel is exposed to an inorganic aqueous solution that is unsaturated in relation to hydroxyapatite and fluorapatite, the enamel surface is altered, by forming macrolesion that is microscopically similar to erosion that develops in the oral cavity (2). This situation may occur clinically when people consume a large quantity of acid drinks, particularly by excessive ingestion of fruit juices and citric flavored carbonated sodas (e.g., orange and lemon) (2,3). The macroscopic appearance of the tooth surface area frequently exposed to these solutions becomes whitened, chalk-like and opaque (2). The chemical factors that make substance erosive to dental tissues and restorative materials present in the oral cavity may be the pH, type of acid, adhesion to the tooth surface, buffer capacity, chelating properties and concentrations of calcium, phosphate and fluoride (3,4).

Erosion is a type of non-carious cervical lesion that develops with consequent loss of tooth structure caused by chemical action, without the involvement of bacteria, and may be of intrinsic or extrinsic origin. The extrinsic causative factors are: diet (fruits, acidic beverages), environment (chemical industries, chlorinated swimming pools) and medications (Vitamin C, asprin, hydrochloric acid) or also idiopathic factors (without established cause). The intrinsic factors are: diseases that cause gastric fluid reflux or reduced salivary flow. Extrinsic factors are frequently observed in daily clinical practice and the frequency of some hygiene habits have been related to the occurrence of tooth erosion (2,4).

Children that consume citric fruits more than twice a day present a 37 times higher risk of developing lesions by erosion than those who do not consume them due to specific characteristics of primary teeth. Similar risks occur with the consumption of apple vinegar (10 times higher) or soft drinks (4 times higher) risk, when consumed on a daily basis. The progression of dental structure loss by erosion maybe approximately 1µm per day (5,6).

Some studies (7,8) have been conducted relative to erosion wear of the dental structure due to indiscriminate consumption of acidic beverages, however few studies have been concerned about investigating the possible erosive effect related to the use of children’s mouth washes on dental restorative materials, such as glass ionomer cement (GIC), commonly used in the clinical practice of pediatric dentistry.

Considering that glass ionomer cement is widely applied in dental clinics for pediatric patients, it has become of the utmost importance to investigate the erosive action of mouthwashes for the pediatric dental public, routinely used in the oral hygiene habits of this population, on this restorative material. Thus, the objective of this present study was to investigate the erosive potential of infant mouthwashes on glass ionomer cement samples by means of roughness analysis after an erosive cycling process.

## Material and Methods

-Study groups

After perform a pilot study, it was considered a chance of 80% to detect a 25% of change difference after erosive cycling between the mouthwashes groups at 5% level of significance, it was required 10 samples of each group to perform the present study. Thus, for this *in vitro* study, 40 test specimens (n=10) were fabricated and subdivided into groups according to the following active agents mouthwashes: G1 - cetylpyridinium chloride (Cepacol Teen – Safoni Aventis Farmacêutica Ltda., Suzano, SP, Brazil), G2 - xylitol and triclosan (Dentalclean Garfield - Rabbit Ind. Com de Prod. de Higiene Pessoal Ltda., Londrina, PR, Brazil) and G3 - Malva sylvestris and xylitol (Malvatrikids Júnior - Daudt Oliveira Ltda., Rio de Janeiro, RJ, Brazil). In addition, distilled water was used as negative control (G4).

-Sample Preparation

A total of 40 round-shaped test specimens (10mm x 2mm) were fabricated of conventional high viscosity glass ionomer cement (Ketac Molar Easymix- 3M ESPE, St. Paul, USA) by using a metal matrix. The disc was filled with glass ionomer after manipulation performed in accordance with the manufacturer’s instructions. Afterwards, a uniform polyester matrix interposed with a glass slide was applied to the specimens. On reaching setting time, the specimens were finished with abrasive discs (Soft Lex 3M ESPE, Sumaré, SP, Brazil) under controlled pressure, and a standardized number of times of application with the purpose of uniformizing the test specimen surfaces (8).

-Mean surface roughness test

The surface roughness of the samples was measured with a rugosimeter (Mitutoyo Corporation, Japan) and the value was expressed as the arithmetic roughness value (Ra = µm). Each sample was carefully dried with absorbent paper before the roughness measurements. The final Ra values of each sample were obtained by the arithmetic mean of three consecutive measurements of each test specimen (initial roughness = Ri). For this step, the ISO 1997 specifications were used, with a cut-off value of 0.8 and speed of 0.5mm/s (8).

-Erosive Cycling Process

The test specimens were immersed in artificial saliva (Saliform, Fórmula e Ação, São Paulo, SP, Brazil) for a period of 24 hours after sample preparation and initial roughness measurement. Subsequently, they were submitted to erosive cycling for 15 days. Each day of cycling consisted of 2 exposure cycles by the mouthwashes under study for 2 minutes, and remineralization in artificial saliva for 10 hours at ambient temperature. At the end of each cycling session, the samples were immersed in new artificial saliva and kept in an oven at 370C for a minimum period of 14 hours (overnight). The negative control was submitted to the same process (distilled water).

-ΔWear Evaluation

**Figure 1:**
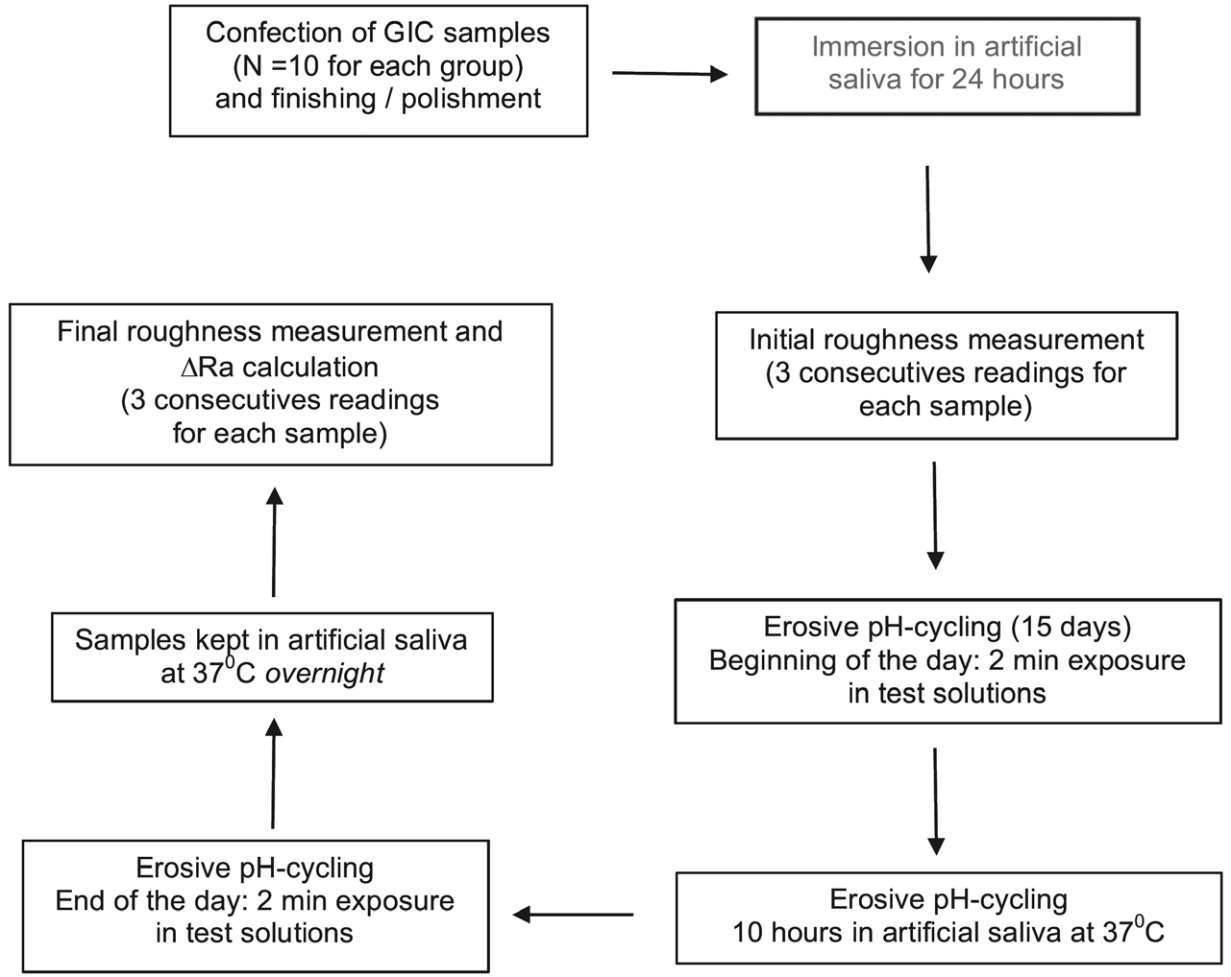
Flow chart of the study design.

At the end of the 15th day of erosive challenge, the test specimens were resubmitted to roughness analysis (final roughness = Rf) to obtain the real increase in roughness (ΔRa). Aiming to clarify the study design, a flow chart was made as follow below, (Fig. 1)

-Statistical Analysis 

As a manner of comparing the erosive potential of the mouth washes the ΔRa values were compared with those of the control group. As the sample presented normal and homogeneous distribution, the ANOVA test was used for analyzing the difference in values of the means obtained among them, followed by the Tukey-test, taking the value of 5% as reference for statistically significant differences. Yet, to verify the mean differences of each group after 15 days of erosive cycling, it was used T-test for dependent samples. The software program SPSS for Windows, version 23.0 (IBM, Armonk, NY, USA) was used for sample size calculation and statistical analysis.

-pH Analysis

Furthermore, the pH (hydrogenionic potential) of the mouthwashes was analyzed. For this purpose, a pH-meter (Hanna Instruments, PA, USA) was used. In which 5 mL of each mouthwash analyzed was transferred to a microcentrifuge tube and by means of a specific electrode for reading the variable, the pH was measured. A valid reminder is that pH is temperature-dependent, therefore, to simulate a mouth-rinse, all the mouthwashes samples presented the same initial temperature, which was maintained at ambient temperature before each measurement.

## Results

The present study data were quantitatively evaluated in a descriptive (pH) and inferential (roughness values) manner. Thus, the data relative to pH measurement are described in  Table 1.

**Table 1:**
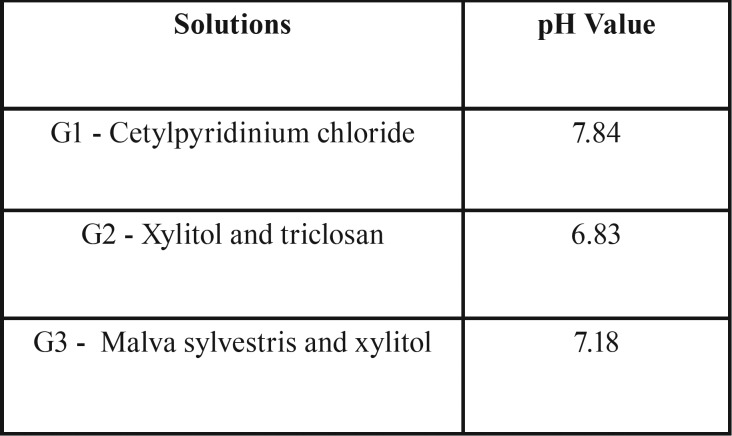
Comparison of mouthwashes pH values.

The triclosan and xylitol-based mouthwash (G2) presented a slightly more acidic pH in comparison with the other mouthwashes tested.

With reference to roughness values analysis obtained are described in  Table 2.

**Table 2:**
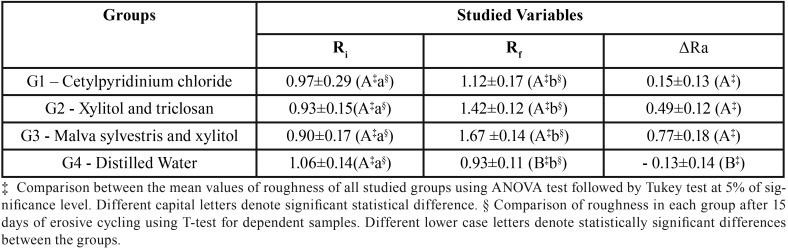
Roughness values analysis of studied groups.

It could be observed that the test samples submitted to exposure to Malva sylvestris and xylitol-based mouthwash (G3) resulted in the highest analyzed values (Rf = 1.67 ± 0.14; ΔRa = 0.77 ± 0.18), followed by the xylitol and triclosan-based mouthwash (G2 - Rf = 1.42 ± 0.12; ΔRa = 0.49 ± 0.12) and the cetylpyridinium chloride-based mouthwash (G1 - Rf =1.12 ± 0.17; ΔRa = 0.15 ± 0.13), which presented statistically significant difference in comparison to the control group (G4 - Rf = 0.93 ± 0.11; ΔRa = -0.13 ± 0.14) both relative to final roughness (Rf) value and real increase in roughness (ΔRa). In addition, it was verified that after 15 days of erosive cycling was an increase of the studied variable to each group, in exception to distilled water, which resulted in a lower final roughness value.

## Discussion

Mouthwashes were introduced in view of the limitations of mechanical oral hygiene methods, and used as complement to this measure that is widely used for biofilm control. To this, substances such as sodium fluoride, cetylpyridinium chloride, triclosan, chlorhexidine, thymol, and tyrothricin, among others may be mentioned (9,10).

In some countries, the majority of mouthwashes are available on the market without requiring a prescription from a dental surgeon, thus making it easy for the general public to buy these products. This fact, added to the situation that manufacturers do not emphasize or warn against abusive use, or consider the correct indications, increase the damages and/or side effects on oral cavity, specially in children. Accordingly, there has been an increase in mouthrinse sales in the UK and usage habits have been arises as well, with some individuals using these rinses up to more than five times daily (11 Pretty). In summary, any solution that is arguably abused than the usual, in this manner predisposes erosion risk, apart of substrate type (e.g. dental or restorative material) (11).

One explanation regarding this issue resides on that some components/properties of mouthwashes. Viscosity, total soluble solids content (TSSC), pH, and calcium/phosphate content must be considered as predisposing factors to influence restorative materials properties (i.e. roughness, hardness and weight), dental counterparts, and soft tissues, such as, tongue and gingiva (12-19).

In the present study, the GIC specimens exposed to infant mouthwashes suffered increase of their roughness values with consequent higher values of ΔRa compared to control group. Specifically to this material, it can infer that the frequent contact to these kind of solutions provides dissolution of hydrogel layer, predisposing to higher solubility and water absorption resulting on matrix degradation, with a negative impact on mechanical properties, such as the tested here (roughness) (20-21).

Relative to material point of view, the long-term contact to mouthwashes was capable of pores creation, and the occurrence of hills, valleys and voids resulting on GIC surface modification as well as manipulation and composition could be some causative factors as well. (22). This same profile could be created by other substances as seen by Karda *et al.* (23) where verified that, between different restorative materials, the highest mean average surface roughness was reached by the GIC group eroded by various acid drinks. Yet, investigation of Briso *et al.* (22) demonstrated that GICs were severely eroded by HCl after several days of storage, whereas Bakar *et al.* (24), using the same acid solutions, experienced greater dissolution at the margin of the edge restoration/teeth, which was confirmed by SEM (scanning electronic microscopy) images. On this same way, Soares *et al.* (25) verified that a GIC submitted to Pepsi Twist and a RMGIC (resin-modified GIC) eroded by Pepsi Twist + a mouthwash containing triclosan + gantrez were found the materials that mineral loss was more expressive attested by energy-dispersive X-ray fluorescence.

In relation to the test period used (15 days), the literature shows a divergence varying from minutes (26), hours (11,27), days (23,28-29) until weeks (22). Despite of some authors attesting that just after long-term use of acidic solutions would be able to be causative of erosion (13,14), Ostrowska *et al.* (29) revealed that after only 1 hour of exposure of orange juice and isotonic beverages were capable of Ra increased values of human teeth samples, attesting that not only materials are damage but also the dental structure could be harmed. On this same way, after a storage of five weeks in HCl and Sprite soft drink, GIC materials such as Fuji II LC and Vitremer had their mean surface roughness 2-folded times (22). Thus, there is a lack of evidence attesting the best protocol to perform this study type, which is in dependence of the primary goal. Thinking on this subject, this investigation followed a similar recent study, using the same study design, but with some pertinent modifications due to test the purposed hypothesis.

In order to simulate the oral cavity and its pH variation along of the day, it was decided to use an dynamic erosive pH-cycling using saliva for remineralization process between the erosive cycles as using it for overnight step as well. Although the majority of studies usually keep the substrates contacted acidic solutions over a prolonged time, they did not account for the important role of saliva, making comparisons to other studies a difficult task. Francisconi *et al.* (21) using a pH-cycling for 7 days concluded no significant differences among restorative materials regarding roughness, but with an increase alteration on ionomeric group. On the other hand, Honorio *et al.* (20) on the same year, using a longer pH erosive cycling (35 days) promoted significant alterations to materials tested, specially on GICs. However, both studies resulted in lower wear values when exposed to saliva only, demonstrating the protective and crucial effects of this substance on challenging situations, permanently faced on oral environment, which was the main focus of this investigation.

The resistance and surface roughness of restorative materials to wear are of great clinical importance as these properties are known to be part of the longevity of restorations in permanent and primary teeth. Since GICs are largely used in clinical practice, it is of some importance investigate its survival rate on population. To this, a recent systematic review and metanalysis was conducted with conclusion that the median survival rate (MSR) of conventional ionomeric restorations presented a lower MSR than RMGIC and compomers in primary molars (30). Thus, any factor that could alter negatively the properties of dental materials, including the question investigated here, represent an alert to guide our clinical practice and postures regarding the use of chemical agents in children, specially those under control of cariogenic factors using ionomer cement restorations.

In summary, it is important to evaluate the risk-benefit produced by these products. In case of dental erosion, the regimen and duration of use of a potentially erosive agent would be critical for the result. Yap *et al.* (8) warned that those products should be used with caution, especially with reference to the concentrations indicated by the manufacturers; frequency and mode of using the mouthwash, because the presence of acid components in their formulations could make the products potentially erosive in the long term. Specifically in relation to the child population, this inadvertent use without prescription and/or correct indication could not only damage the primary dental structure, which has a lower volume of tooth enamel, but may also have a negative influence on the surface characteristics of ionomer cement that was the target of the present study and risk of microbiological unbalance as well.

Therefore, the authors concluded that the children’s mouth washes tested in the present study, used in an erosive cycling model, increased the surface roughness of glass ionomer cement test specimens.
